# *Trypanosoma brucei* BRCA2 acts in antigenic variation and has undergone a recent expansion in BRC repeat number that is important during homologous recombination

**DOI:** 10.1111/j.1365-2958.2008.06230.x

**Published:** 2008-06

**Authors:** Claire L Hartley, Richard McCulloch

**Affiliations:** The Wellcome Centre for Molecular Parasitology and Faculty of Biomedical and Life Sciences, University of Glasgow, Glasgow Biomedical Research Centre 120 University Place, Glasgow G12 8TA, UK

## Abstract

Antigenic variation in *Trypanosoma brucei* has selected for the evolution of a massive archive of silent Variant Surface Glycoprotein (*VSG*) genes, which are activated by recombination into specialized expression sites. Such *VSG* switching can occur at rates substantially higher than background mutation and is dependent on homologous recombination, a core DNA repair reaction. A key regulator of homologous recombination is BRCA2, a protein that binds RAD51, the enzyme responsible for DNA strand exchange. Here, we show that *T. brucei* BRCA2 has undergone a recent, striking expansion in the number of BRC repeats, a sequence element that mediates interaction with RAD51. *T. brucei* BRCA2 mutants are shown to be significantly impaired in antigenic variation and display genome instability. By generating BRCA2 variants with reduced BRC repeat numbers, we show that the BRC expansion is crucial in determining the efficiency of *T. brucei* homologous recombination and RAD51 localization. Remarkably, however, this appears not to be a major determinant of the activation of at least some *VSG* genes.

## Introduction

African trypanosomes, in common with many pathogens, survive in the face of host immune responses by undergoing antigenic variation, the continuous changing of pathogen surface antigens. Successive waves of immune response to the variant surface antigens eradicate part but not all of the pathogen population, lengthening the infection and enhancing transmission. A number of strategies for antigenic variation have evolved (Deitsch *et al.*, 1997), but a common approach is the use of gene conversion (Palmer and Brayton, 2007). *Trypanosoma brucei* antigenic variation involves changes in the composition of a dense, protective coat composed of Variant Surface Glycoprotein (VSG) and can occur at very high rates (Turner and Barry, 1989), a trait shared with other pathogens (Barry and McCulloch, 2001; Criss *et al.*, 2005). *T. brucei* appears remarkable, however, in the extent to which it has invested in gene conversion (for recent reviews, see Horn and Barry, 2005; Taylor and Rudenko, 2006).

The *T. brucei* genome contains greater than 1000 *VSG* genes (Berriman *et al.*, 2005; Marcello and Barry, 2007), an archive of potential variant surface proteins larger than in any other organism described to date. The genome is composed of 100 or so minichromosomes, a few intermediate chromosomes and 11 megabase chromosomes. *VSG*s are found adjacent to the telomere on all *T. brucei* chromosome classes, but the majority of the archive is present in subtelomeric arrays on the megabase chromosomes. Each trypanosome cell normally expresses only one VSG at a time, from telomeric *VSG* expression sites (ESs). The main route of VSG switching is the movement of the silent *VSG* genes into the ESs (Robinson *et al.*, 1999). Primarily, such recombination reactions involve gene conversion, generating a copy of a silent *VSG* and displacing the ES *VSG*. Less common are cross-overs in which chromosome ends are exchanged, activating a new *VSG* without copying. *VSG* gene conversions appear to operate in a hierarchy, which is thought to be important in extending the infection (Pays, 1989; Barbet and Kamper, 1993). Telomere-proximal *VSG* genes are activated early, followed by functional array genes (Morrison *et al.*, 2005). Finally, novel mosaic *VSG*s are generated by segmental gene conversion reactions involving *VSG* pseudogenes and gene fragments (Thon *et al.*, 1990; Kamper and Barbet, 1992). Such damaged *VSG*s represent the huge majority of the *VSG* archive, and are likely to be a major determinant of the function and success of *T. brucei* antigenic variation (Kamper and Barbet, 1992; Barbet and Kamper, 1993; Marcello and Barry, 2007). In contrast to a number of other pathogens, where a single expression locus for antigenic variation is found (Criss *et al.*, 2005; Palmer and Brayton, 2007), *T. brucei* possesses multiple *VSG* ESs (Becker *et al.*, 2004). Only one ES is fully transcribed at a time, but VSG switches can occur by inactivating the active ES and activating a previously silent ES, though the factors that mediate this process are still being determined (Pays, 2005; Hughes *et al.*, 2007).

Homologous recombination performs core functions in all cells by reversing genotoxic damage and ensuring the completion of DNA replication (Sung and Klein, 2006). It can also provide for telomere maintenance, and is needed for genetic exchange during meiosis. Homologous recombination appears to act in *T. brucei VSG* switching, as mutation of RAD51 (McCulloch and Barry, 1999), the central eukaryotic enzyme of homologous strand exchange, impairs the process, as does mutation of a RAD51-related protein, RAD51-3 (Proudfoot and McCulloch, 2005). Such a reliance on a core DNA repair process for the specialized recombination of antigenic variation is true also for *Neisseria* sp. (Sechman *et al.*, 2005). However, whether specific factors for antigenic variation exist, and whether antigenic variation has imposed adaptations on the homologous recombination machinery, have been little explored. One stage at which such features might be seen is in the regulation of RAD51 function, which is influenced by a wide range of factors (Sung and Klein, 2006). One of these is BRCA2, the protein encoded by one human breast cancer susceptibility gene (Narod and Foulkes, 2004). BRCA2 homologues are widespread in eukaryotes (Warren *et al.*, 2002; Lo *et al.*, 2003), and analyses in the nematode *Caenorhabditis elegans* (Petalcorin *et al.*, 2007) (CeBRC-2), in the fungus *Ustilago maydis* (Kojic *et al.*, 2002; Yang *et al.*, 2005) (Brh2) and in the plant *Arabidopsis thaliana* (Dray *et al.*, 2006) suggest that at least one function is conserved with a role initially described in mammals: RAD51 interaction via BRC repeat motifs in BRCA2 (Bork *et al.*, 1996; Wong *et al.*, 1997; Chen *et al.*, 1998; Marmorstein *et al.*, 1998). At least one BRC repeat has been described in all characterized BRCA2 homologues (Lo *et al.*, 2003). Mammalian BRCA2 has eight non-identical BRC repeats (Lo *et al.*, 2003), at least six of which bind RAD51 in humans (Wong *et al.*, 1997; Chen *et al.*, 1998; Marmorstein *et al.*, 1998). Similarly, *A. thaliana* BRCA2 has four degenerate BRC motifs, some of which bind RAD51 (Dray *et al.*, 2006). In contrast, *C. elegans* BRCA2/CeBRC-2 (Martin *et al.*, 2005) and *U. maydis* BRCA2/Brh2(Kojic *et al.*, 2006) contain only a single RAD51-binding BRC repeat. In addition to binding RAD51 via the BRC repeats, BRCA2 interacts with RAD51 through unrelated sequences in a number of organisms (Mizuta *et al.*, 1997; Sharan *et al.*, 1997; Esashi *et al.*, 2005; Petalcorin *et al.*, 2007; Zhou *et al.*, 2007). In mammals and *C. elegans*, non-BRC repeat binding appears to be specific for RAD51 filaments (Davies and Pellegrini, 2007; Esashi *et al.*, 2007; Petalcorin *et al.*, 2007), whereas the BRC repeats can bind both RAD51 monomers and filaments (Galkin *et al.*, 2005; Shivji *et al.*, 2006).

Previous work has suggested that a BRCA2 homologue in *T. brucei* contains a remarkable expansion in BRC repeat number (Warren *et al.*, 2002; Lo *et al.*, 2003). Here, we have examined BRCA2 from *T. brucei* and related kinetoplastid parasites, and show that this is a recent evolutionary adaptation. To test if this is due to the use of homologous recombination in antigenic variation, we have generated *T. brucei* BRCA2 mutants and variants of the protein with alterations in conserved motifs, including the BRC repeats. We show, first, that BRCA2 acts in DNA repair, recombination and antigenic variation in *T. brucei*. Second, we demonstrate that the BRC repeat number or sequence composition of BRCA2 is critical in general repair and recombination but, remarkably, appears not to be important in the activation of early expressed *VSG* genes.

## Results

### A recent evolutionary expansion of BRC repeats in *T. brucei* BRCA2

**Fig. 2:**
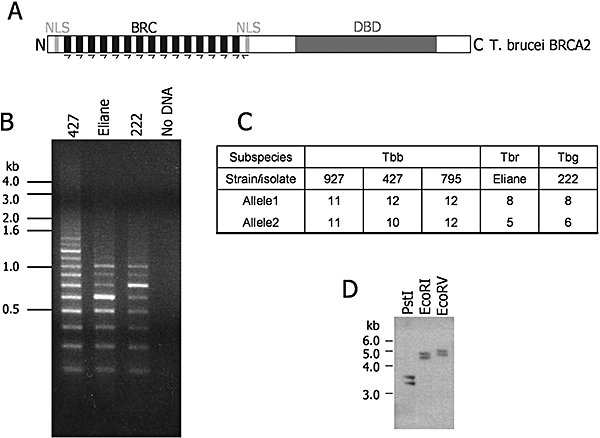
Analysis of BRC repeat number in *T. brucei* BRCA2. A. The predicted domain organization of BRCA2 from *T. b. brucei* strain TREU927 is shown; 15 putative BRC repeats are indicated, as is the DSS1-DNA-binding domain (DBD) and two putative nuclear localization signals (NLSs). Arrows denote oligonucleotide primers used in minisatellite variant repeat (MVR) mapping. B. MVR mapping of BRC repeat number in genomic DNA from *T. b. brucei* strain Lister 427, *T. b. rhodesiense* isolate Eliane and *T. b. gambiense* isolate 222; size markers are indicated. C. A summary of the numbers of BRC repeats in the two alleles of *BRCA2* in *T. b. brucei* (Tbb) strains TREU927, Lister 427 and EATRO795, and in isolates of the *T. brucei* subspecies *T. b. rhodesiense* (Tbr; Eliane) and *T. b. gambiense* (Tbg; 222); BRC numbers were inferred from MVR mapping (above), Southern analysis and sequencing of PCR clones of the BRC repeat regions. D. A Southern blot of restriction-digested genomic DNA from *T. b. brucei* strain Lister 427, probed with the *BRCA2* ORF.

*Trypanosoma brucei* and related, evolutionarily diverged kinetoplastid parasites each encode a single BRCA2 homologue (Warren *et al.*, 2002; Lo *et al.*, 2003). These proteins possess significant sequence similarity with BRCA2 homologues from vertebrates, plants and fungi in the DSS1-DNA binding region (Lo *et al.*, 2003), within which most domains are conserved (Yang *et al.*, 2002; C. Hartley and R. McCulloch, submitted). Regulation of the kinetoplastid BRCA2 proteins' function by DSS1 is also likely to be conserved, as a homologue of DSS1 is also identifiable in each kinetoplastid genome and the BRCA2–DSS1 interacting residues (Yang *et al.*, 2002) are conserved in each protein. Finally, it seems likely that the kinetoplast BRCA2 proteins are nuclear, as localization signals can be detected in the *T. brucei* (Fig. 2A) and *T. cruzi* polypeptide sequences (data not shown).

**Fig. 1:**
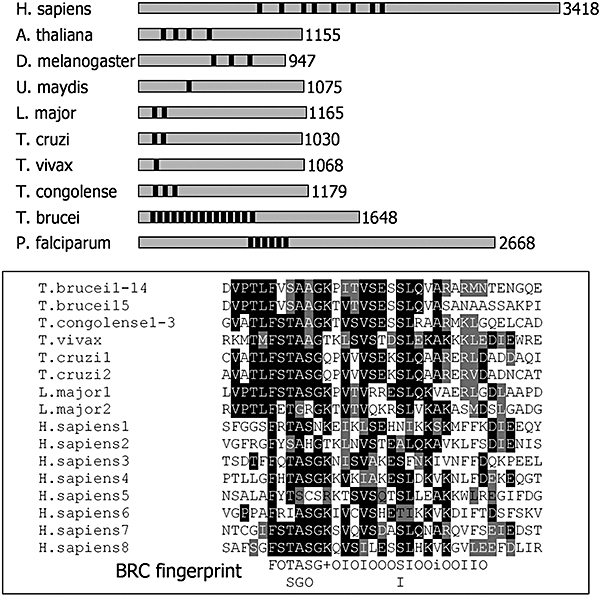
Conservation of BRC repeats in eukaryotic BRCA2. The upper diagrams show the number and location of BRC repeat motifs (black bars) in BRCA2 proteins from a number of eukaryotes; the sizes of the BRCA2 polypeptides (in amino acids) are indicated. The boxed diagram shows conservation of BRC repeats in trypanosomatid BRCA2 proteins relative to *H. sapiens*; critical residues for RAD51 binding inferred by Lo *et al.* (2003) are indicated as a BRC fingerprint (O, polar; +, positively charged; I, hydrophobic; i, small hydrophobic).

The only other clear similarity between kinetoplastid and other eukaryotic BRCA2 proteins lies in the BRC repeats (Bork *et al.*, 1996; Bignell *et al.*, 1997). However, the BRC repeat number and organization in *T. brucei* are highly unusual (Fig. 1 and Table S1). In general terms, it appears that the simpler the organism, the smaller the number of BRC repeats in BRCA2. In illustration, of 14 BRCA2 proteins identified in the genomes of a diverse range of unicellular organisms (Table S1), nine have between one and three BRC repeats. Conversely, of eight multicellular organisms, seven have three or more BRC repeats: most vertebrate BRCA2 proteins contain eight repeats, the plant *A. thaliana* has four, and the insects *Drosophila melanogaster* and *Anopheles gambiae* have three and 10 respectively. It seems plausible therefore that developmental complexity and/or increased genome size exerts an evolutionary pressure for greater numbers of repeats due to greater demands for control of homologous recombination. *C. elegans* BRCA2/CeBRC-2 appears to be an exception, however, with only one BRC repeat, although the reasons for this are unclear. Some protists are also exceptions, displaying greater BRC repeat numbers. *Trichomonas vaginalis* BRCA2 has 14 predicted BRC repeats, which may be related to the large genome size of this parasite (Carlton *et al.*, 2007). However, this does not always provide an explanation, as Apicomplexan BRCA2 proteins, such as in *Cryptosporidium parvum*, *Plasmodium falciparum* and *Toxoplasma gondii*, have between six and eight predicted BRC repeats and *T. brucei* BRCA2 is predicted to contain 15.

The BRC repeats of *T. brucei* BRCA2 are unusual in two further ways. First, unlike the broad conservation of increased BRC repeat number in the apicomplexans, BRCA2 of *T. brucei* is distinct from closely related kinetoplastids (Fig. 1). *T. cruzi* and *Leishmania major* diverged from *T. brucei* 200–300 million years ago (Overath *et al.*, 2001) and each BRCA2 has two non-identical BRC repeats. Even more strikingly, two *Trypanosoma* species, *T. vivax* and *T. congolense*, that belong to the same salivarian clade as *T. brucei* (Cortez *et al.*, 2006) are predicted to possess BRCA2 proteins with only one and three BRC repeats respectively. The second unusual feature of *T. brucei* BRCA2 is that the 15 predicted BRC repeats are arranged as a tandem array in which 14 are identical in sequence [in fact, each repeat of the array is 44 amino acids (132 bp) in length, longer than the 35-amino-acid BRC motif; Fig. 1]. In all other organisms in which BRCA2 has more than one BRC repeat, with the notable exception of *T. congolense*, they are distributed unevenly in the polypeptide sequence and are degenerate in sequence outwith the key functional residues (Lo *et al.*, 2003).

Before examining why *T. brucei* BRCA2 has such unusual structural organization, we checked that the above predictions do not arise from genome sequence compilation errors. We first performed minisatellite variant repeat (MVR) mapping on clones of a number of *T. b. brucei* strains and *T. brucei* subspecies (Fig. 2). In all cases, MVR PCR yielded the expected ladder of products. In *T. b. brucei* strain Lister 427, the largest product was equivalent to 12 BRC repeats and a pronounced, smaller band was generated with 10 repeats, suggesting two distinct *BRCA2* alleles (Fig. 2A). In the *T. b. brucei* genome sequence strain TREU927, which was predicted to have 15 BRC repeats, each allele contained 11 repeats, while a further *T. b. brucei* strain, EATRO795, contained two alleles with 12 BRC repeats (Fig. 2C). MVR PCR of isolates from *T. b. rhodesiense* and *T. b. gambiense* suggested a lower number of BRC repeats than in *T. b. brucei*: each contained a larger allele with eight repeats and a smaller allele with five or six repeats respectively (Fig. 2B and C). To confirm these findings, we performed Southern mapping of *BRCA2*, demonstrating two distinct *BRCA2* alleles of the expected size in both *T. b. brucei* Lister 427 (Fig. 2D) and *T. b. gambiense* (data not shown). Next, we PCR-amplified the BRC-repeat region from *BRCA2* from each of the above *T. brucei* strains and subspecies, as well as from *T. vivax* and *T. congolense*, and sequenced the products after cloning. For *T. brucei*, this confirmed the predicted BRC repeat organization: in all cases, the most C-terminal coding repeat (‘BRC repeat 15’, Fig. 1) was degenerate in sequence relative to all the upstream repeats, which were virtually identical at the nucleotide level (< 1 base change per repeat, on average) both within a given *BRCA2* gene and between strains/subspecies (data not shown). *BRCA2* from *T. vivax* was confirmed as containing a single BRC repeat, while the *T. congolense* strain used here (TREU1457) contained two very similar (5 bp differences) repeats, rather than the three predicted from genome sequencing (strain IL3000). Taken together, these data indicate that the BRC repeat organization in *T. brucei* BRCA2 is a recent evolutionary expansion, generating numbers that, while variable between isolates, are significantly higher than *BRCA2* orthologues in closely related kinetoplastid parasites.

### *T. brucei* BRCA2 acts in homologous recombination and antigenic variation

**Fig. 3:**
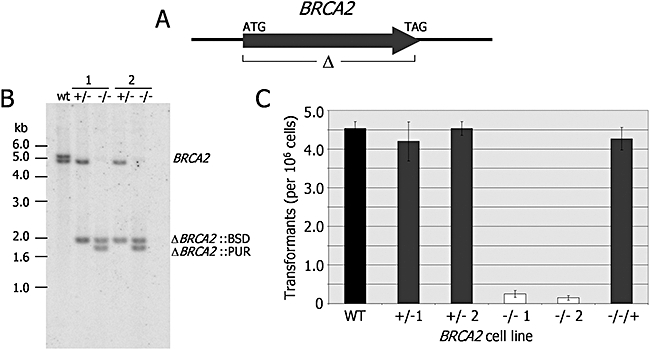
Assaying the role of *T. brucei* BRCA2 in homologous recombination. A. The complete *BRCA2* ORF was deleted (triangle) in wild type (WT) bloodstream stage *T. brucei* cells and replaced by cassettes encoding resistance to blasticidin (Δ*BRCA2*::*BSD*) or puromycin (Δ*BRCA2*::*PUR*). B. A Southern blot of SacII- and StuI-digested genomic DNA from WT cells (two distinct *BRCA2* alleles can be seen), and from two independently generated heterozygous (+/−1, 2) and homozygous mutants (−/− 1, 2). Size markers (kb) and the positions of intact and disrupted *BRCA2* alleles are indicated. C. To assay recombination, the number of transformants recovered (per 10^6^ cells put on antibiotic selection) when the construct tub-*HYG*-tub was electroporated into the parasite cell lines was measured; values shown are averages from three experiments, and bars indicate standard errors. BRCA2 re-expressers (−/−/+) were generated by re-inserting the *BRCA2* ORF into one of the −/− mutants.

To examine the functions of BRCA2 in *T. brucei* we generated null mutants in bloodstream stage cells, deleting the entire *BRCA2* ORF (Fig. 3) by two rounds of transformation, replacing the two alleles with constructs encoding resistance to blasticidin and puromycin. This was performed in both the Lister 427 *T. brucei* strain and in a transgenic derivative named 3174 (McCulloch *et al.*, 1997; McCulloch and Barry, 1999; Proudfoot and McCulloch, 2005), which allows analysis of switching mechanisms. In each strain, we independently derived two lines of *BRCA2* heterozygous (+/−) and homozygous (−/−) mutants, and re-expressed BRCA2 in one of the −/− mutants (−/−/+). In all cases, the mutants (Fig. 3B) and re-expressers were confirmed by Southern mapping and reverse transcription polymerase chain reaction (RT-PCR) (data not shown). Consistent with a role for BRCA2 in DNA repair, the −/− mutants displayed increased sensitivity to methyl methanesulphonate (MMS) or bleomycin-induced DNA damage relative to wild type (WT), +/− or −/−/+ cells (C. Hartley and R. McCulloch, submitted). In addition, the *brca2*−/− mutants displayed replication or cell division defects not observed in mutants of other *T. brucei* DNA repair and homologous recombination genes (C. Hartley and R. McCulloch, submitted).

To examine the contribution of BRCA2 to *T. brucei* homologous recombination, the transformation efficiency of WT, +/−, −/− and −/−/+ cells was compared following electroporation with targeting constructs. In the Lister 427 mutants, we used linearized tub-*HYG*-tub plasmid, which targets a hygromycin resistance gene to the tubulin array, replacing an α tubulin ORF by homologous recombination on terminal flanking sequence (240 bp of tubulin β–α intergenic sequence upstream, and 330 bp of α–β intergenic sequence downstream). Transformants were generated in the *brca2*−/− mutants 12.5- to 22.5-fold less efficiently than the other cells (Fig. 3C). Very similar results were found in 3174 mutants using an equivalent plasmid in which *HYG* was replaced by a phleomycin resistance gene (data not shown). In all cases, examples of the *brca2*−/− transformants were characterized by Southern blotting, which showed that all integrations had targeted the tubulin array via homology (data not shown). These results are comparable with mutants of other *T. brucei* homologous recombination factors (RAD51, RAD51-3, RAD51-5 and MRE11) (McCulloch and Barry, 1999; Conway *et al.*, 2002; Robinson *et al.*, 2002; Proudfoot and McCulloch, 2005) and indicate that BRCA2 acts in homologous plasmid integration, but BRCA2-independent pathways also operate.

**Fig. 4:**
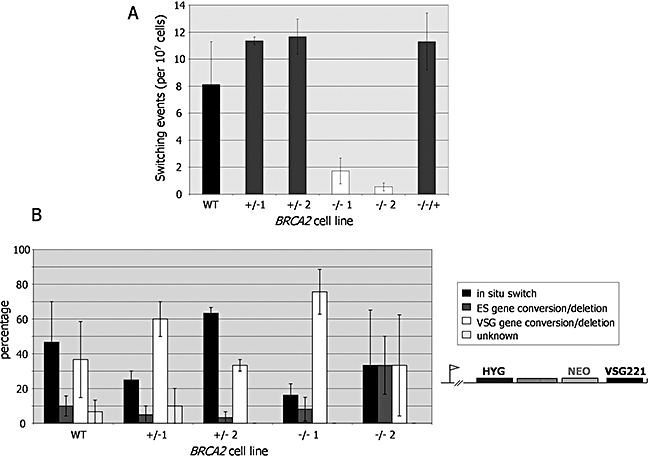
Assaying the role of *T. brucei* BRCA2 in VSG switching. A. VSG-switching frequencies for wild-type *T. brucei* cells (3174.2; WT) were compared with two independently generated heterozygous (+/−1, 2) and homozygous (−/− 1, 2) BRCA2 mutants, in addition to a cell line in which BRCA2 was re-expressed from the tubulin locus (−/−/+). The values shown are the means of at least three independent experiments, and bars represent standard errors. B. The mechanism of VSG switching used in each cell line was determined by assaying for the expression of hygromycin and G418 resistance from the antibiotic marker genes (*HYG* and *NEO* respectively) inserted around *VSG*-associated 70 bp repeat sequences (hatched box) in the active *VSG221* expression site (ES) in strain 3174 (diagram shows this gene arrangement; The flag denotes promoter), as well as using PCR to assess whether *HYG*, *NEO* or *VSG221* had been removed by gene conversion or DNA deletion. The percentage of clones that had undergone VSG switching by transcriptional (*in situ*) switching, gene conversion/deletion of *HYG*, *NEO* and *VSG221* (ES gene conversion/deletion) or gene conversion/deletion of only *NEO* and *VSG221* (VSG gene conversion/deletion) for each cell line is shown; bars denote standard errors, and switched variants that do not conform to the above mechanisms are indicated as ‘unknown’.

We next used the mutant cell lines made in the 3174 strain to examine the ability of the *brca2*−/− mutants to undergo antigenic variation. Measuring the efficiency at which VSG-switched variants arose (Fig. 4A) indicated that ablation of *BRCA2* reduced the VSG-switching frequency around 8- to 11-fold relative to the WT, +/− or −/−/+ cells. The extent of this switching impairment is very similar to that seen in *RAD51* and *RAD51-3* mutants (McCulloch and Barry, 1999; Proudfoot and McCulloch, 2005), confirming that much of VSG switching involves homologous recombination. Characterization of the reactions that gave rise to VSG-switched variants indicated that gene conversion and transcriptional switching events could still be detected in the *brca2*−/− cells (Fig. 4B), indicating that the reduced VSG switch frequency was accounted for by impairment of both reaction pathways. This surprising result is equivalent to VSG-switching phenotypes observed in *RAD51* and *RAD51-3* mutants (McCulloch and Barry, 1999; Proudfoot and McCulloch, 2005) and may indicate that BRCA2 acts in conjunction with these factors in this process.

### The BRC repeat organization of *T. brucei* BRCA2 is an important determinant of homologous recombination efficiency

**Fig. 5:**
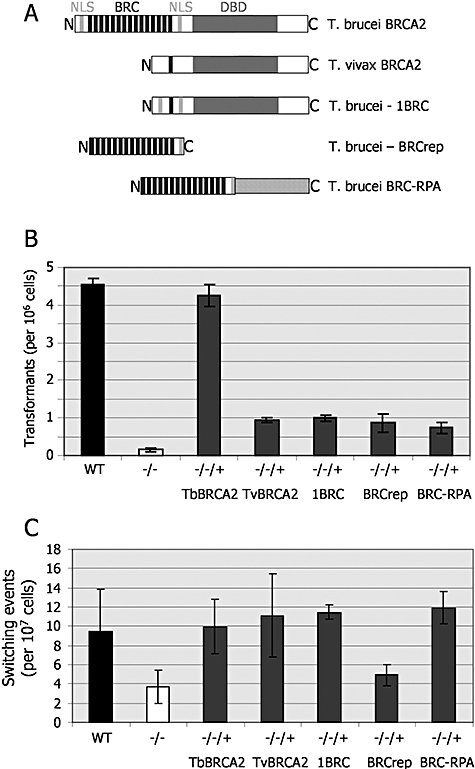
BRCA2 variants and polypeptide fusions to assess the function of the BRC repeats in *T. brucei* recombination and VSG switching. A. Functional domains of BRCA2 variants and polypeptide fusions analysed by expression in bloodstream stage *brca2*−/− mutants are shown. Full-length *T. brucei* BRCA2 may contain up to 15 BRC repeats, a conserved DSS1-DNA-binding domain (DBD) and two putative nuclear localization signals (NLSs). *T. vivax* BRCA2 displays conservation of the DBD, but has only one BRC repeat and NLSs have not been predicted. 1BRC differs from full-length *T. brucei* BRCA2 only in reduction of the BRC array to a single repeat. BRCrep is a polypeptide fragment of *T. brucei* BRCA2 encompassing the BRC repeats and 33 downstream amino acids, including a bipartite NLS. BRC–RPA is a fusion of the BRCrep polypeptide to the 50 kDa *T. brucei* replication protein A subunit. B. Homologous recombination efficiency was assayed by determining the number of transformants recovered (per 10^6^ cells put on antibiotic selection) when the construct tub-*HYG*-tub was electroporated into wild-type (WT) cells, *brca2*−/− mutants and −/− cells expressing the BRCA2 variant polypeptides detailed above (−/−/+). C. VSG-switching frequencies of WT (Lister 427) *T. brucei* cells, *brca2*−/− mutants and −/− cells expressing the BRCA2 variant polypeptides are shown. Values are the means of at least three independent experiments, and bars represent standard error.

To examine why *T. brucei* BRCA2 has evolved such an unusual arrangement of BRC repeats, we generated a number of BRCA2 variants (Fig. 5A). To test if BRCA2 with a single BRC repeat is competent in DNA repair and recombination we examined BRCA2 from *T.vivax* (TvBRCA2), which naturally possesses a single BRC repeat (Fig. 1). TvBRCA2 and *T. brucei* BRCA2 (TbBRCA2) share 26% overall sequence identity, rising to 42% C-terminal to the BRC repeats. Sequence comparisons predict that TvBRCA2 will bind *T. brucei* RAD51: the BRC repeat retains all the key residues for RAD51 interaction predicted by Lo *et al.* (2003) (Fig. 1); and the single RAD51 orthologue in each *Trypanosoma* species share 79% sequence identity (87% similarity) with each other, including conservation of all residues predicted to bind BRC repeats (data not shown) (Lo *et al.*, 2003). We also manipulated the *T. brucei BRCA2* gene, reducing the BRC repeat number to just one. In this protein (1BRC) only the variant, C-terminal BRC repeat (‘repeat 15’; Fig. 1) is retained, but all other parts of BRCA2 are unaltered. Despite being distinct from the upstream repeats, the C-terminal BRC peptide retains residues predicted to be critical for binding RAD51 (Lo *et al.*, 2003). Next, a truncated version of *T. brucei BRCA2* was generated (BRCrep) that encodes only the BRC repeat region plus a C-terminal predicted nuclear localization signal (NLS); all C-terminal sequence (including the DSS1-DNA-binding domain) was deleted, as was 80-amino-acid N-terminal to the BRC repeats. Finally, a *BRCA2* variant was generated in which the BRCrep polypeptide was translationally fused to the *T. brucei* Replication Protein A (RPA) 50 kDa subunit, which is homologous to the 70 kDa RPA-1 protein of other eukaryotes and binds single-stranded DNA via a conserved oligonucleotide/oligosaccharide binding fold domain in *Leishmania* (Neto *et al.*, 2007). This BRC–RPA fusion is functionally equivalent to similar polypeptides examined in *U. maydis* (Kojic *et al.*, 2005) and mammalian cells (Saeki *et al.*, 2006).

Each of the above variants, plus full-length TbBRCA2, were expressed in one of the *brca2*−/− Lister 427 cell lines by integration of the ORFs into the *T. brucei tubulin* array. All were expressed using the same integration construct, meaning that their level of expression, assuming equal translation efficiencies, should be comparable and correct integration was confirmed by Southern analysis (data not shown). Expression of most of the proteins could be demonstrated by complementation of phenotypic defects: BRC–RPA and TbBRCA2 reverted the MMS sensitivity of the *brca2*−/− cells (C. Hartley and R. McCulloch, submitted), TbBRCA2, TvBRCA2 and 1BRC reverted the replication/cell division defect of the mutants (C. Hartley and R. McCulloch, submitted) and all but BRCrep was active in VSG switching (see below). To examine if the BRCA2 variants function in homologous recombination, the transformation efficiency of tub-*HYG*-tub was compared in the different expresser cell lines (Fig. 5B). As we have shown already (Fig. 3), full-length TbBRCA2 caused reversion of the *brca2*−/− integration defect, with transformation frequencies equivalent to WT and +/− cells. In contrast, none of the variant proteins functioned efficiently in this assay. Although transformants arose in each expresser cell line at slightly higher rates than −/− cells, in each case this was significantly lower (around 4–5) than the frequencies seen in the WT or TbBRCA2 −/−/+ cell lines (*P*-values < 0.001 for all variants; two-sample *t*-test). This indicates that each is impaired in this pathway of homologous recombination.

### *T. brucei* BRCA2 with a single BRC repeat is impaired in RAD51 focus formation

**Fig. 6:**
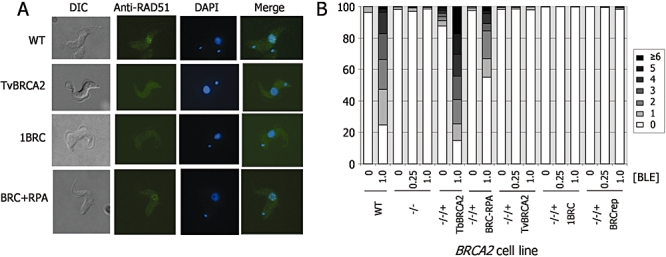
RAD51 subnuclear foci in *T. brucei* bloodstream stage cells. A. An example of a wild-type (WT) *T. brucei* cell with discernible subnuclear RAD51 foci following 18 h growth in 1.0 μg ml^−1^ phleomycin is shown (top). Below are examples of *brca2*−/− cells expressing either *T. vivax* BRCA2, *T. brucei* BRCA2 with only one BRC repeat (1BRC) or a fusion of the *T. brucei* BRC repeats of BRCA2 and RPA following the same treatment. Cells were visualized by differential interference contrast (DIC; left), the DNA was stained with DAPI (right centre) and RAD51 (left centre) was visualized by indirect immunofluorescence. Merged DAPI and RAD51 images are also shown (right). B. Quantification of the number of RAD51 foci detectable after 18 h growth in differing concentrations of phleomycin (BLE; μg ml^−1^); BRCA2 variants expressed in the *brca2*−/− cells (indicated by −/−/+) are detailed in Fig. 5.

Phleomycin treatment and induction of a DNA double-strand break in *T. brucei* causes re-localization of RAD51 to discrete subnuclear foci (Proudfoot and McCulloch, 2005; Glover *et al.*, 2008). Formation of such foci appears to be a conserved response to DNA damage that is controlled by a number of factors (see Lisby and Rothstein, 2004; Tarsounas *et al.*, 2004 for reviews), including BRCA2 in other eukaryotes (Tarsounas *et al.*, 2003; Yu *et al.*, 2003; Kojic *et al.*, 2005; Martin *et al.*, 2005). To examine if this is true also in *T. brucei*, we examined RAD51 localization by indirect immunofluorescence, before and after phleomycin treatment, in the BRCA2 mutants (Fig. 6). As we have described before, very few RAD51 foci were detectable in the absence of damage (only around 3–10% of cells), whereas treatment with 1.0 μg ml^−1^phleomycin for 18 h resulted in detectable foci in > 75% of WT, +/− and full-length TbBRCA2 −/−/+ cells (Fig. 6B). In contrast, no evidence was found for RAD51 foci induction in the *brca2*−/− cells, suggesting that BRCA2 has a profound role in this response (Fig. 6B). The −/− cells in which either TvBRCA2, the 1BRC variant or the BRCrep truncation was expressed displayed a similar deficiency in RAD51 foci formation (Fig. 6). In contrast, BRC–RPA was capable of supporting foci formation (Fig. 6A), despite being impaired in homologous integration. Quantification of the response of the −/−/+ BRC–RPA cells showed they were, in fact, somewhat less efficient at making RAD51 foci than WT cells (Fig. 6B), which correlates the partially increased sensitivity to phleomycin we have observed (C. Hartley and R. McCulloch, submitted). Nevertheless, as has been described in mammalian cells (Saeki *et al.*, 2006) and in *U. maydis* (Kojic *et al.*, 2005), a fusion of the *T. brucei* BRC repeats with a heterologous DNA-binding domain is capable of functioning in DNA damage repair. Why, then, the *T. brucei* BRC–RPA fusion is non-functional in homologous recombination, unlike the equivalent protein in mammalian cells (Saeki *et al.*, 2006), is not clear. This may reflect differences in the assays used in Saeki *et al.* (2006) compared with here, or distinct activities of the parasite and host BRCA2 proteins.

### *T. brucei* BRCA2 with a single BRC repeat is functional in VSG switching

To examine if the BRCA2 variants support VSG switching, we used the expresser cell lines in Lister 427, rather than generating equivalent cell lines in strain 3174, thereby allowing direct comparison with the phenotypic analyses discussed above. Strain 3174 is useful in analysing VSG switching as it contains antibiotic resistance markers in the active VSG ES (encoding VSG221) (McCulloch *et al.*, 1997), meaning that singular VSG expression in a population can be selected by antibiotic pressure, and switched variants allowed to arise over a defined number of generations by removing that selection. The antibiotic markers can also be used to characterize the switching events that gave rise to clonal switched variants, which are recovered by immune selection *in vivo*. Lister 427 also expresses the *VSG221* ES, but it is not genetically marked, so exclusive expression cannot be maintained and examination of VSG switching is limited to measuring VSG-switching frequency. Before analysing VSG switching, we first confirmed, by Western analysis, that VSG221 continued to be expressed in each expresser cell line (data not shown). We then followed an abridged version of the strain 3174 VSG-switching assay described previously (McCulloch *et al.*, 1997; McCulloch and Barry, 1999). Although there was greater variation, the VSG-switching frequency determined for WT Lister 427 cells (Fig. 5C) was comparable with WT 3174 (see Fig. 4), and the *brca2*−/− mutants were still seen to be impaired (Fig. 5C). Strikingly, both cell lines expressing a BRCA2 variant with a single BRC repeat, and the cells expressing the BRC–RPA fusion, were unaltered in their capacity to undergo VSG switching relative to either the WT cells or the −/−/+ TbBRCA2 cells (Fig. 5C). Only the cells expressing the isolated BRC repeat domain were impaired in VSG switching. These results indicate that the tandem array of identical BRC repeats in *T. brucei* BRCA2, despite being crucial for efficient plasmid integration, re-localization of RAD51 following phleomycin damage and DNA repair (C. Hartley and R. McCulloch, unpublished), is not a determinant of VSG switching efficiency during an acute infection.

### *T. brucei* BRCA2 mutants display gross chromosomal rearrangements in their megabase chromosomes

**Fig. 7:**
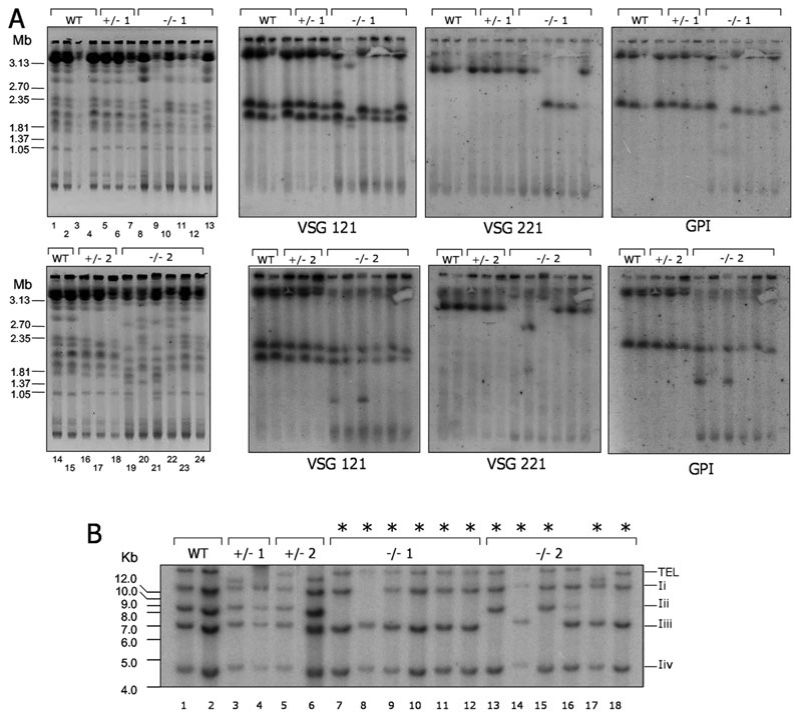
Karyotype changes in BRCA2 mutants. A. The top and bottom panels to the left show ethidium bromide-stained PFG electrophoretic separations of intact chromosomes from clones of wild-type (WT) cells and from two independently generated BRCA2 heterozygous (+/−) and homozygous (−/−) mutants that had been grown *in vitro* for ∼290 generations; size markers (Mb) are shown, and lane numbers are indicated. To the right of this are three Southern blots in which the PFGs were probed, successively, with *VSG121*, *VSG221* and glucose 6-phosphate isomerase (*GPI*). B. A Southern blot of XmnI-digested genomic DNA from samples of the WT, +/− and −/− mutant clones (above). The DNA was separated on a 0.8% agarose gel and the blot probed with *VSG121*, of which one telomeric (TEL) and four putative subtelomeric array copies are present (Ii–iv). Size markers (kb) and lane numbers are indicated, and clones in which at least one gene copy had been deleted are indicated by asterisks.

BRCA2 mutation in mammalian cells causes accumulation of aberrant chromosomes, including chromosome loss, breakage and translocations (Patel *et al.*, 1998; Yu *et al.*, 2000). Similar gross chromosomal rearrangements (GCRs) have been described in *U. maydis* BRCA2/Brh2 mutants (Kojic *et al.*, 2002). To examine this in *T. brucei*, each of the +/− and −/−*BRCA2* cells lines, as well as WT cells, were cloned and grown for ∼290 generations *in vitro*. The cells were then re-cloned, and the karyotypes of a number of clones were analysed by Pulsed Field Gel (PFG) electrophoresis (Fig. 7A). The megabase chromosomes of *T. brucei* range in size from ∼1 to 6 Mb in Lister 427 strain (Melville *et al.*, 2000), and in this gel separation the greatest size discrimination is seen from ∼1 to 2.5 Mb. Considerable variation in chromosome size between *brca2*−/− clones, indicative of GCR, was apparent even from ethidium bromide staining (particularly mutant 2), in contrast to the relative stability in the +/− or WT clones. However, closer examination suggests two things. First, most such GCR involved reduction in chromosome size. Second, a trend towards reduction in chromosome size, though not as severe as the −/− clones, was also apparent in the *BRCA2*+/− cells, perhaps indicative of haploinsufficiency. Probing the PFGs with *VSG121* (a five-gene family), *VSG221* (a single-copy *VSG* in the active *VSG* ES) or *GPI* (a single-copy gene encoding glucose 6-phosphate isomerase) appears to confirm these findings. Only in two clones (lanes 19 and 21), when probed with *VSG221*, did we find evidence for an increase in chromosome size. *VSG121* hybridizes to two chromosomes of approximately 2.1 and 2.3 Mb, and both appear to be smaller in all the *brca2*−/− clones relative to the WT cells (by as much as 100 kb in some cases). The same chromosomes also appear to have become smaller in two to three of the five *BRCA2*+/− clones, though to a lesser extent. The *VSG221* and *GPI* blots show that the extent of size change in the chromosomes of the *brca2*−/− cells can be very severe (for instance, around 500 kb in three *brca2*−/− clones probed with *VSG221*). In addition, the smaller homologue of chromosome 1 that harbours *GPI* (around 2.3 Mb) appears to have reduced in size in three to four of the five *BRCA2*+/− clones. A Southern blot of XmnI-digested genomic DNA from all of the above clones, probed with *VSG121*, confirms that the chromosomal changes can involve loss of genetic material, as 11 of the 12 *brca2*−/− clones had lost at least one of the five gene copies (Fig. 7B). Notably, in no case was the telomeric copy lost, despite the gene being silent in this strain. Indeed, all clones continued to express *VSG221* (data not shown), consistent with telomeric sequences being relatively stable.

**Fig. 8:**
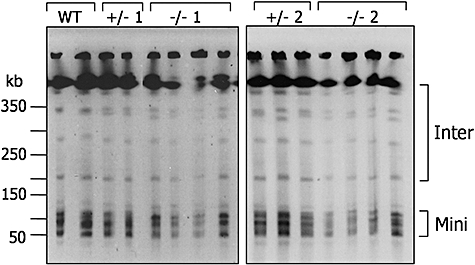
*T. brucei* intermediate and minichromosomes in BRCA2 mutants. The panels show ethidium bromide-stained PFG electrophoretic separations of intermediate chromosomes (Inter) and minichromosomes (Mini) from clones of wild-type (WT) cells and from two independently generated BRCA2 heterozygous (+/−) and homozygous (−/−) mutants that had been grown *in vitro* for ∼290 generations; size markers (kb) are shown.

Remarkably, the same GCRs were not observed in karyotyping of either the intermediate or minichromosomes (Fig. 8). For both these classes of chromosome, which contain primarily *VSG* and *VSG* ES sequences as the main genetic material that can be expressed, the karyotype was substantially more stable than the megabase chromosomes in the *brca2*−/− mutants, with no severe differences in chromosome size detectable compared with the *BRCA2*+/− and WT cells. This indicates that although BRCA2 mutation in *T. brucei* results in GCRs, this is limited, at least predominantly, to the megabase chromosomes.

## Discussion

In this report we have characterized a homologue of BRCA2 in the parasite *T. brucei* and document the function of the protein in the maintenance of genome stability, in recombination and in antigenic variation. Although BRCA2 is widely conserved in the eukaryotic kingdom (Warren *et al.*, 2002; Lo *et al.*, 2003), *T. brucei* is arguably the most diverged eukaryote in which its cellular functions have been examined. The structural organization of *T. brucei* BRCA2 is highly unusual, possessing an expansion in the number of BRC repeat motifs, which are critical in the interaction of BRCA2 with the key enzyme of eukaryotic homologous recombination, RAD51 (Pellegrini and Venkitaraman, 2004; Shivji and Venkitaraman, 2004; Sung and Klein, 2006). Most characterized single-celled eukaryotes encode BRCA2 homologues with one to three BRC repeats, in contrast with the majority of multicellular eukaryotes, where BRCA2 normally has three or more BRC repeats (Lo *et al.*, 2003). The BRC repeat number in *T. brucei* BRCA2 is variable between different strains and subspecies of the parasite, but is always between three- and sixfold greater than found in most single-celled organisms (with the exception of apicomplexans and *T. vaginalis*; see below), and in some strains is greater than has been described anywhere else in nature. Given that this expansion appears not to be present in closely related kinetoplastid organisms, and that the BRC repeat organization takes the form of a tandem array of repeats that are virtually identical in sequence (which contrasts with the dispersed, diverged repeats found in most other organisms) this appears to be a recent evolutionary adaptation. The high-sequence homology of the BRC repeats most likely results in ongoing array expansion and contraction, explaining the variation in repeat number we have documented.

No work to date has examined why some BRCA2 molecules require multiple BRC repeats while others function with as few as one repeat. The simplest explanation is that, in general, the evolution of eukaryotes with increasing genome size and developmental complexity requires greater control or greater overall activity of homologous recombination, which is reflected in more extensive interactions between BRCA2 and RAD51. For example, a larger number of BRC motifs might be needed to sequester the putatively greater amount of RAD51 until it is needed for repair, perhaps preventing uncontrolled recombination. This would be compatible with the demonstration that the BRC repeats can disrupt the RAD51 filament, block its assembly and impair recombination, and infers that BRCA2 can maintain RAD51 as a monomer via the BRC repeats (Chen *et al.*, 1999; Davies *et al.*, 2001; Pellegrini *et al.*, 2002; Shin *et al.*, 2003). Indeed, studies of GFP-labelled RAD51 have suggested that some RAD51 exists in the mammalian nucleus in relatively immobile pools, one of which is BRCA2-bound, as well as in another pool of relatively greater mobility (Essers *et al.*, 2002; Yu *et al.*, 2003). Alternatively, the number of BRC repeats may vary to ensure that sufficient RAD51 is available at the site of DNA double strand breaks, the abundance of which might be dependent on genome size and/or sequence complexity (Pellegrini *et al.*, 2002). Such an explanation implies that BRCA2–RAD51 interaction involves more than just sequestration, for which there is accumulating evidence. It is now clear that RAD51 binding to BRCA2 through non-BRC sequences in both mammals (Davies and Pellegrini, 2007; Esashi *et al.*, 2007) and *C. elegans* (Petalcorin *et al.*, 2007) is specific for, and stabilizes, RAD51 filaments. *In vitro* studies have also shown that isolated BRC motifs can, in some conditions, bind RAD51 filaments without causing disruption (Galkin *et al.*, 2005), and that a polypeptide from *Homo sapiens* BRCA2 spanning all eight BRC repeats can promote RAD51 strand exchange (Shivji *et al.*, 2006). Furthermore, a fusion of the BRC repeats from either *H. sapiens* (Saeki *et al.*, 2006) or *U. maydis* (Kojic *et al.*, 2005) BRCA2 with RPA, thereby excluding non-BRC-RAD51 binding motifs, also functions in DNA repair and recombination. These data indicate that BRC-RAD51 binding can play an active role in RAD51 recombination, perhaps during the delivery of RAD51 to sites of damage through BRCA2 binding to single-strand DNA tails (Yang *et al.*, 2005).

The evolutionary pressures that have dictated the expansion of BRC repeat numbers in *T. brucei* are unknown, but could either reflect specific aspects of the biology of this parasite or reveal general aspects of BRCA2 function. The hypothesis we sought to test is that the BRC repeat expansion is a consequence of antigenic variation. VSG switching is, at least in part, reliant on RAD51-dependent homologous recombination, as mutation of RAD51 impairs the process (McCulloch and Barry, 1999), as does mutation of the RAD51 paralogue RAD51-3 (Proudfoot and McCulloch, 2005). The work here adds to that picture by showing that BRCA2 is also critical for efficient VSG switching, as *brca2*−/− mutants are significantly impaired in this reaction. It should be noted that the impairment of VSG switching in all these DNA repair mutants appears to affect the activation of new *VSG*s both by gene conversion and by transcriptional switching between *VSG* ESs, questioning whether these reactions are enzymatically and mechanistically distinct (Proudfoot and McCulloch, 2005). Irrespective of this, antigenic variation can occur at rates measured to be substantially greater than background mutation (Turner and Barry, 1989), are primarily driven by gene conversion (Robinson *et al.*, 1999) and involve the activation of *VSG*s from an archive of silent genes numbering > 1000 and found dispersed throughout the subtelomeres of most, if not all, *T. brucei* chromosomes (Barry *et al.*, 2005; Marcello and Barry, 2007). Any, and perhaps all, of these factors might explain why BRCA2 in *T. brucei* has evolved such a specialized structure. However, we find that BRCA2 with only one BRC repeat, either retaining only the divergent C-terminal repeat or being BRCA2 from *T. vivax*, is capable of supporting efficient VSG switching. This suggests that the expanded BRC repeat number of *T. brucei* BRCA2 is not a consequence of antigenic variation, with two important caveats. First, we have examined VSG switching in Lister 427, a monomorphic strain that undergoes VSG switching at rates of only around 1 × 10^−6^ switches per cell per generation (see Figs 4 and 8), less than the pleomorphic cells in which high switching rates (up to 1 × 10^−2^) were measured. However, the BRC repeat number of BRCA2 in Lister 427 is among the highest we characterized, and BRC numbers vary considerably in *T. brucei*, which appears to suggest that the selective pressures that expanded the BRC array are retained in low-switching strains. Second, our assay measures VSG switching only very early in an infection, when telomere-proximal, intact *VSG* genes are predominantly activated (Pays, 1989; Morrison *et al.*, 2005; Marcello and Barry, 2007). Later in infections *VSG* pseudogenes and gene fragments become the preferred substrates for switching (Thon *et al.*, 1990; Marcello and Barry, 2007) and it is therefore possible that *T. brucei* BRCA2 structure reflects distinctive features of these reactions.

The finding that VSG switching may not be the basis for the unusual structure of *T. brucei* BRCA2 has further support. *T. congolense* and *T. vivax* are closely related to *T. brucei* and also survive in mammals through antigenic variation of a VSG surface coat, which in *T. congolense* has been shown to involve gene duplication (Majiwa *et al.*, 1985). Despite this, the BRCA2 orthologues of these species possess two to three and one BRC repeats, respectively, which is more typical of single-celled eukaryotes. It is worth noting, however, that the genome annotation of *T. congolense BRCA2* predicts three highly related BRC repeats present in an array, perhaps indicating an abridged version of the BRC expansion in *T. brucei*. It will be interesting to see how the size and organization of the *VSG* system in these parasites relate to *T. brucei*. Nevertheless, BRC repeat expansion in protists is not limited to *T. brucei*. Previous work showed that BRCA2 homologues in *Plasmodium* have six BRC repeats (Lo *et al.*, 2003), and we now show that this may be a common feature of Apicomplexans. Furthermore, *T. vaginalis* BRCA2 appears to possess 14 BRC repeats. Whether or not the BRCA2 organization in these parasites shares a functional basis with *T. brucei* is unknown.

Our data illustrate that, whatever the basis for the selection of BRC repeat expansion, this structural organization of *T. brucei* BRCA2 is critical for general repair and recombination. BRCA2 with a single repeat does not support efficient homologous recombination and impairs the ability of the cell to repair both MMS and phleomycin-induced DNA damage (C. Hartley and R. McCulloch, submitted). Most likely, this reflects impaired BRCA2 interaction with RAD51, as visible re-localization of RAD51 to subnuclear foci following phleomyin damage was only seen in parasites expressing full-length TbBRCA2 or BRC–RPA. Why, then, can BRCA2 with only one BRC support efficient VSG switching? A number of possibilities might be considered. The first is that general repair/recombination and antigenic variation have differing requirements, the former involving extensive interaction between BRCA2 and RAD51 through a large number of BRC repeats and the latter needing less extensive interaction. A second possibility is that the BRC repeat organization of *T. brucei* BRCA2 represents a dichotomy in function between the majority, conserved repeats and the downstream divergent repeat: for instance, the former class may provide interactions with RAD51 interaction that directs general repair/recombination, while the latter have evolved for a specific form of RAD51 interaction during antigenic variation. For either of these scenarios to be true, antigenic variation in *T. vivax* and *T. congolense* must differ from that of *T. brucei* and such a distinction from general repair/recombination must be absent. A final possibility therefore is that the *T. vivax* BRCA2 protein and the *T. brucei* variant retaining only the divergent C-terminal BRC repeat do not interact with *T. brucei* RAD51 sufficiently to mediate repair/recombination. If correct, the ability of these proteins to support VSG switching is perplexing, but may suggest that the reaction involved (which is mediated by RAD51, RAD51-3 and BRCA2) has significant mechanistic distinctions from general recombination pathways in the parasite. In this light, it is worth noting that two further proteins that act in general repair and recombination in *T. brucei*, RAD51-5 and MRE11 (Robinson *et al.*, 2002; Proudfoot and McCulloch, 2005), appear not to influence VSG switching.

Further work will be required to evaluate the above hypotheses. Examining the unusual BRC repeat expansion of *T. brucei* BRCA2 could shed light on both the biochemistry of recombination during antigenic variation and the evolution of BRCA2 for recombination in general. For instance, it is intriguing that the GCRs found in the *T. brucei brca2*−/− mutants were very similar to those described previously in mutants of MRE11 (Robinson *et al.*, 2002). In each case the chromosome size changes were primarily due to sequences loss, telomeric elements appeared to be relatively unaffected and rearrangements were only clearly seen in the megabase chromosomes. It is tempting to speculate that this indicates a shared function of the proteins in maintenance or use of the subtelomeric *VSG* arrays, but it is also possible that this reflects broadly conserved roles in genome stability.

## Experimental procedures

### Assaying recombination and VSG switching

*Trypanosoma brucei* Lister 427 bloodstream cells of strains MITat1.2a and 3174 (McCulloch *et al.*, 1997) were grown at 37°C in HMI-9 medium (Hirumi and Hirumi, 1989) and transformed by electroporation as described previously (Conway *et al.*, 2002; Bell and McCulloch, 2003). Details of the generation of BRCA2 mutants are described elsewhere (C. Hartley and R. McCulloch, submitted). To assay recombination, 5 × 10^7^cells of each cell line, grown to a maximum of 2 × 10^6^ cells ml^−1^, were transformed with 5 μg of XhoI- and XbaI-digested tub-*HYG*-tub, recovered in non-selective media for three generations (taking account of growth rate) and transformants were selected with 5 μg ml^−1^ hygromycin. For WT and +/− cells, 5 × 10^6^ electroporated cells were plated over 24 wells in 1.5 ml well^−1^, whereas 2 × 10^7^−/− cells were plated over 48 wells. VSG-switching frequencies in 3174 cell lines were determined as described previously (McCulloch and Barry, 1999; Proudfoot and McCulloch, 2005; 2006). For the Lister 427 MITat1.2a cell lines, essentially the same procedure was used, but with some modifications. At least three mice were infected with 2 × 10^5^cells of each BRCA2 variant cell line, and the infections cured by cymelarsan treatment after the parasitaemia reached > 5 × 10^7^ cells ml^−1^ of blood, generating mice immune to the VSGs expressed. To select for switched variants, 4 or 8 × 10^7^ cells of each BRCA2 variant cell line (growing *in vitro*) were inoculated into a mouse previously infected with the same variant, and recovered 24 h later by exsanguination. The number of switched variants in the blood was measured as described previously (McCulloch and Barry, 1999; Proudfoot and McCulloch, 2005; 2006). Details of the *BRCA2* variant constructs and targeting are described elsewhere (C. Hartley and R. McCulloch, submitted).

### MVR mapping

For each *T. brucei* strain or subspecies, the *BRCA2* ORF was PCR-amplified from genomic DNA with Taq DNA Polymerase (ABgene) and the primers TbBRCA2for (5′-ATGAGCCACAAAAAAGGAAGACAAGGC) and TbBRCA2rev (5′-TTCTCGCATAAGATCAGCG) to provide a substrate for MVR PCR reactions, which contained 5 μM primers TbBRCrepfor (5′-GCGGTACAAGGAAATTCCAC) and TbBRCreprev (5′-AGGAGGCACCTGCTCCCGAA) and 5 U of Taq DNA polymerase (ABgene). MVR PCR was performed in 75 mM Tris-HCl (pH 8.8), 20 mM (NH_4_)_2_SO_4_, 0.01% (v/v) Tween-20 and 1.5 mM MgCl_2_ for 18, 21 or 28 cycles of 95°C for 1 min, 55°C for 1 min and 72°C for 4 min, and the products were separated by 1.5% agarose gel electrophoresis.

### RAD51 immunofluoresence

*Trypanosoma brucei* in culture were harvested by centrifugation at 583 *g* for 10 min at room temperature, washed in PBS and re-suspended in 100 μl of PBS. One millilitre of 1% (v/v) formaldehyde in PBS was added and the sample incubated at 4°C for 1 h. The cells were then centrifuged at 6000 *g* for 1 min, washed twice in chilled PBS, washed once in 500 μl of chilled 1% BSA in H_2_O, re-suspended in 30 μl of the same solution and smeared across a slide and allowed to air dry for 3 h. After re-hydration in PBS for 5 min, the slides were blocked by adding 50% fetal bovine serum in PBS (FBS/PBS) for 15 min. Blocking solution was then removed and the slides were transferred to a dark, humid chamber before adding rabbit polyclonal anti-TbRAD51 antibody (Proudfoot and McCulloch, 2005) diluted 1:500 in 3% FBS/PBS for 45 min. The slides were then washed twice in PBS for 5 min, returned to the humid chamber and Alexa 488-conjugated goat-derived anti-rabbit IgG, diluted 1:1000 in 3% FBS/PBS, added and incubated for 45 min at room temperature. Finally, the slides were washed twice in PBS for 5 min, allowed to air dry, then mounted in Vectashield with DAPI (4,6-diamidino-2-phenylindole) (Vector Laboratories). Fluorescence microscopic analysis was performed using an Axioskop 2 microscope (Zeiss) and images were obtained with Openlab software (Improvision).

### PFG electrophoresis

Agarose plugs containing genomic DNA from 5 × 10^7^ cells were prepared as described previously (Barnes and McCulloch, 2007). A CHEF-DR III (Bio-Rad) apparatus was used for electrophoretic separation. 1× TB1/10E (90 mM Tris base, 90 mM boric acid, 2 mM EDTA) and 1.2% agarose (Seakem LE, BioWhittaker Molecular Applications) was used for the separation of megabase chromosomes, whereas 0.5× TBE (45 mM Tris base, 45 mM boric acid, 10 mM EDTA) and 1.0% agarose was used for the separation of smaller chromosomes. Agarose plugs were prepared by three rounds of dialysis in the appropriate electrophoresis buffer. Gels were electrophoresed at 15°C, either at 2.5 V cm^−1^ for 144 h with an initial switch time of 1400 s and final switch time of 700 s for separation of megabase chromosomes, or 5.8 V cm^−1^ for 24 h with initial and final switch times of 20 s for separation of smaller chromosomes. DNA was visualized by UV illumination following staining of the gels in 0.2 μg ml^−1^ ethidium bromide. Southern blots were prepared by capillary transfer onto hybond-XL membrane (Amersham), and were probed with [α-^32^P]-labelled DNA generated by random priming and washed to 0.2× SSC, 0.1% SDS at 65°C.

## Supplementary material

This material is available as part of the online article from: http://www.blackwell-synergy.com/doi/abs/10.1111/j.1365-2958.2008.06230.x (This link will take you to the article abstract).
