# Supplementary material for: Trypanosoma brucei BRCA2 acts in antigenic variation and has undergone a recent expansion in BRC repeat number that is important during homologous recombination
Source: Mol Microbiol. 2008 Jun;68(5):1237–51. doi: 10.1111/j.1365-2958.2008.06230.x (PMC2408642; doi:10.1111/j.1365-2958.2008.06230.x)
Supplement: Supplementary file 1 [file mmi0068-1237-SD1.pdf]

Taxon	Organism	Status of genome project	Size (amino acids)	No of BRC repeats
Entamoebidae	*E. histolytica*	Complete	719	1
Parabasala	*T. vaginalis*	Complete	1664	14
Diplomonadida	*G. lamblia*	Complete	1104	1
Euglenozoa	*T. brucei*	Complete	1648	15
	T. vivax	5X coverage	1068	1
	T. congolense	1X coverage	1179	3
	T. cruzi	Complete	1030	2
	L. major	Complete	1165	2
Apicomplexa	*C. parvum*	Complete	1442	8
	P. falciparum	Complete	2668	6
	T. gondii	10X coverage	2741	8
Viridiplantae	*A. thaliana*	Complete	1155	4
Mycetozoa	*D. discoideum*	Complete	1623	1
Microsporidia	*E. cuniculi*	Complete	490	1
Fungi	*U. maydis*	Complete	1075	1
Metazoa	*C. elegans*	Complete	394	1
	A. gambiae	Complete	2037	10
	D. melanogaster	Complete	971	3
	C. familiaris	Complete	3446	8
	F. catus	2X coverage	3372	8
	H. sapiens	Complete	3418	8
	G. gallus	Complete	3397	8

Supplementary Table 1. The number of BRC repeats in BRCA2 proteins (total polypeptide size in amino acids is shown) from a number of eukaryotic organisms, covering a number of taxons, is indicated; whether or not the genomes have been sequenced to completion or near completion (estimated fold coverage) is shown. NCBI accession numbers: *E. histolytica*, EAL46403; *T. vaginalis*, EAY04622; *G. lamblia*, EDO79616; *T. brucei*, CAJ15962; *L. major*, CAJ03514; *T. cruzi*, EAN94204; *C. parvum*, EAK89641; *A. thaliana*, NP_195783; *D. discoideum*, XP_629153; *E. cuniculi*, NP_585972; *U. maydis*, AAM92489; *C. elegans*, AAR98640; *A. gambiae*, XP_001237134; *D. melanogaster*, NP_611925; *C. familiaris*, BAB91245; *F. catus*, BAC75821; *H. sapiens*, P51587; *G. gallus*, AAL89470. Genome sequence database identifiers: *T. vivax*, tviv192h02.q1k_9 (GeneDB); *T. congolense*, congo695a05.p1k_18 (GeneDB); *T. gondii*, 49.m03334 (ApiDB); *P. falciparum*, PF13_0155 (ApiDB).
